# Spaced Seed Data Structures for *De Novo* Assembly

**DOI:** 10.1155/2015/196591

**Published:** 2015-10-11

**Authors:** Inanç Birol, Justin Chu, Hamid Mohamadi, Shaun D. Jackman, Karthika Raghavan, Benjamin P. Vandervalk, Anthony Raymond, René L. Warren

**Affiliations:** Canada's Michael Smith Genome Sciences Centre, British Columbia Cancer Agency, Vancouver, BC, Canada V5Z 4S6

## Abstract

*De novo* assembly of the genome of a species is essential in the absence of a reference genome sequence. Many scalable assembly algorithms use the de Bruijn graph (DBG) paradigm to reconstruct genomes, where a table of subsequences of a certain length is derived from the reads, and their overlaps are analyzed to assemble sequences. Despite longer subsequences unlocking longer genomic features for assembly, associated increase in compute resources limits the practicability of DBG over other assembly archetypes already designed for longer reads. Here, we revisit the DBG paradigm to adapt it to the changing sequencing technology landscape and introduce three data structure designs for spaced seeds in the form of paired subsequences. These data structures address memory and run time constraints imposed by longer reads. We observe that when a fixed distance separates seed pairs, it provides increased sequence specificity with increased gap length. Further, we note that Bloom filters would be suitable to implicitly store spaced seeds and be tolerant to sequencing errors. Building on this concept, we describe a data structure for tracking the frequencies of observed spaced seeds. These data structure designs will have applications in genome, transcriptome and metagenome assemblies, and read error correction.

## 1. Introduction

For nearly a century, progressive discovery of the number and molecular structure of chromosomes and their information content have proven to be useful in the clinical domain [1, 2]. With the sequencing of the human genome, we have gained a reference for base pair resolution comparisons that have provided unprecedented insights in molecular and cellular biology. Complementing this reference, development of high throughput sequencing (HTS) platforms, most notably from Roche 454 (Basel, Switzerland), Illumina (San Diego, CA), Life Technologies (Carlsbad, CA), and Pacific Biosciences (Menlo Park, CA), significantly benefited clinical genomics [3, 4], cancer genomics in particular [5, 6]. And there is increased anticipation in the field towards a new sequencing platform from Oxford Nanopore Technologies (Cambridge, UK).

Rapid improvements in the quality and quantity of sequencing data generated by HTS platforms have called for innovative and robust bioinformatics tools. The introduction of read alignment algorithms that use advanced computing science concepts, such as Burrows-Wheeler transformation [7], Ferragina Manzini (FM) indexing [8], and cache oblivious algorithms, allowed the reference-based assembly approach to scale with the exploding volume of HTS data [9–11]. While earlier comparative genomics tools concentrated mostly on analysis of aligned reads [12, 13], the approach biased analyses toward reaffirmation of the reference, even when there is an alternative and parsimonious interpretation.

The fundamental drawback of the reference-based assembly approaches is the consideration of read data independently, ignoring that they are sampling a common underlying sequence. This becomes especially pronounced when a region is highly rearranged, expressed in an unannotated structure, or represented erroneously in the reference. To extend the utility of the reference-based assembly paradigm, several groups developed alternative alignment postprocessing approaches, such as base quality recalibration followed by realignment [14, 15], local assembly with constraints to gain base pair resolution [16–18], or developed methods that measure statistics about putative events, often foregoing base pair resolution [19, 20].

Recently, analysis of HTS data using* de novo* assembly, an approach that is unbiased by the reference sequence, is gaining interest [21–23]. Even though the approach is substantially more computationally intensive, the enhanced specificity and the resulting savings in event verification efforts justify the choice. In earlier work, we had reported on a scalable* de novo* assembly tool, ABySS, that used short reads from an HTS platform to assemble the human genome [24], and we further demonstrated the utility of the approach to analyze transcriptome sequencing (RNA-seq) data (Trans-ABySS) [25, 26]. The technology proved to be valuable in large-scale cancer cohort studies [5, 27–29].

Sequence assembly tools differ in the way they identify read overlaps and disambiguate unclear sequence extensions. A de Bruijn graph (DBG) representation of *k*-mer overlaps (overlaps between sequences of *k* base pairs in length) was introduced with the Euler algorithm [30] and is the enabling technology behind ABySS and some of the other popular* de novo* assembly tools, such as Velvet [31].

The concept hinges on loading *k*-mers into the computer memory to perform fast sequence extension queries. For large target genomes and/or datasets with high sequencing error rate, memory requirement for representing *k-*mers might be prohibitive. ABySS solves this problem by distributing the memory load over a given number of computer nodes in a computer cluster. Minia [32] implements a Bloom filter data structure [33] to represent a DBG stochastically in small memory and navigates it using a secondary data structure.

In sequence assembly, there are several advantages of using a DBG approach compared to overlap-layout-consensus [34] or string graph based assembly algorithms [35]. The former approach uses less memory and executes faster compared to the latter two. However, with increasing read lengths in “short read” platforms like Illumina and with the gaining popularity and development of “long read” platforms like Pacific Biosciences and Oxford Nanopore, DBG based assembly algorithms need to adapt to retain their advantage.

Merely increasing the length of *k-*mers has several problems. For both deterministic (as in ABySS [24]) and stochastic (as in Minia [32]) representation, longer *k*-mers will quickly inflate the memory requirements of the assembly tools, as the experimental data will present a *k-*mer spectrum of increasing volume. Doing so would further result in missed sequence overlaps in the presence of mismatched bases.

The data structure reported in this paper offers a design that will be suitable for extending the utility of fast and effective DBG algorithms, hereby modifying the concept of *k*-mers by introduction of* spaced seeds*. As well, we describe primary and auxiliary data structures based on Bloom filters [33] with potential uses in genome, transcriptome and metagenome assemblies, and error correction.

## 2. Spaced Seeds

Longer reads from technological advances in sequencing platforms and sample preparation coupled with data preprocessing methods (such as the Illumina synthetic long reads) will certainly be a welcome development for assembly-based analysis. However, they also pose certain challenges, most notably due to an increase in the memory required when using longer *k*-mer lengths.

The maximum *k*-mer length that ABySS can use is a compile-time parameter. Currently, we routinely use *k*-mers as long as 120 bp when assembling 2 × 150 bp reads. However, we need to, for instance, increase the number of CPU cores we use for a typical human genome assembly from 36 cores to 48 cores when we increase the *k*-mer length from 96 bp to 120 bp.

Further, a DBG approach assumes that one has error-free *k*-mers, which becomes a strong assumption when the *k*-mer lengths increase, especially with the established long read technology from Pacific Biosciences typically producing reads with error rates of over 10%. Even though the Illumina platforms are generally producing good quality reads with less than 1% average error rate, with increased read lengths the probability of having an error-free *k*-mer still decreases geometrically.

To address both of these issues, we propose using DBGs with spaced seeds. The concept is similar to the paired DBG approach in the Pathset algorithm [36]. The difference is that assembly by spaced seeds would allow for a fixed distance between the seed pairs, as opposed to an undetermined (yet constrained) distance in the Pathset algorithm, which is sensitive to read coverage fluctuations.

For spaced seeds, we use two *k*-mers [*k* : *k*], separated by a fixed distance, Δ. This construct is a special case of the spaced seeds that are used for read alignments [37], where a sequence to be aligned is masked by a template of “match” and “do not care” positions. The alignment process tolerates mismatches in the do not care positions, as long as the match positions agree between the query and the target. In our case, match positions are evenly split and pushed to the 3′ and 5′ ends of the template, and the do not care positions are collected between the flanking match positions.

**Figure 1:**
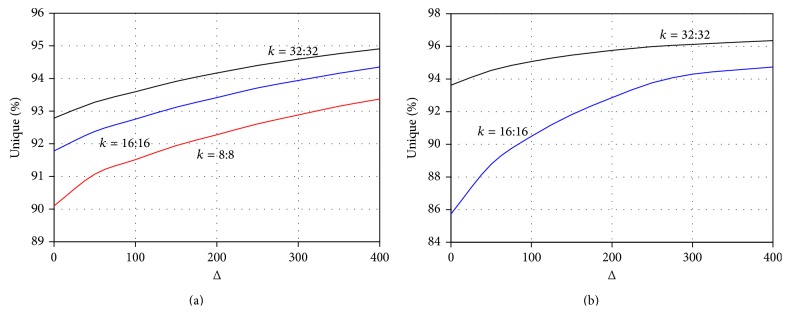
Uniqueness of spaced seeds in the (a)* E. coli* and (b)* H. sapiens* genomes, as a function of the space length. The red, blue, and black curves correspond to spaced seeds of lengths 8, 16, and 32 bp, respectively. When the space length is zero, the uniqueness figures correspond to 16, 32, and 64 bp single *k*-mer lengths, respectively. Curves show that, for the* E. coli* genome, using a spaced seeds of length 16 is equivalent to or better than using *k*-mers of length 64, when delta is longer than 100 bp.

We note that, as the distance between the two *k*-mers increases, the uniqueness of spaced seeds also increases, as demonstrated in Figure 1 for both the* E. coli* and* H. sapiens *genomes. Although this observation is anecdotal, it is compelling that the behavior is so similar for these two species with substantial phylogenetic distance. Our conjecture is that the curves we present in Figure 1 are representative of a wide variety of genomes. This allows one to use spaced seeds to achieve unique representations that are otherwise not possible with single *k*-mers of length 2*k*, thus reducing the memory requirement for assemblies with longer reads. Such a construct would also be tolerant to sequence errors that fall within the space between the seeds.

## 3. Data Structures

### 3.1. Spaced Seeds Hash Table

A straightforward implementation of the data structure within ABySS is possible through modification of the *k*-mer hash table of the software. A hashed *k*-mer holds several pieces of information:

(1) In two-bit base encoding, the sequence content for the first observation of the *k*-mer or its reverse-complement: 2*k* bits.
(2) Frequency of the forward and reverse-complement observations, both maxing out at 2^15^–30 bits (round up to 32 bits).
(3) In the input dataset, presence (1) or absence (0) of all four possible one-base extensions of the sequence in both directions: 8 bits.
(4) Book keeping flags to track *k*-mers removed by error removal algorithms: 16 bits.

For a spaced seeds hash table, we modified (1) to represent the sequence content of the concatenated sequence [*k* : *k*], while applying the same encoding. Of importance, the memory footprint of the spaced seeds is a constant, 4*k*, for a fixed *k*-mer length, and does not depend on the distance between the seeds.

We also modified the sequence extension information to reflect possible extensions of either seed in either direction. This increases the memory footprint of this information to 16 bits.

The data structure for tracking spaced seed frequencies and the book keeping flags are not modified.

Overall, compared to storing a sequence of length (2*k* + Δ), a spaced seed represents savings in memory when Δ > 8.

### 3.2. Spaced Seeds Bloom Filter

In the Bloom filter data structure, a number of hash functions are used to convert a sequence to a large integer value. This value specifies a unique coordinate in allocated memory, when modulus of the calculated integer is used to fit into a predefined memory size. The Bloom filter data structure offers a frugal representation of the set of reads and is typically used to query set memberships. Because of the information loss during hashing and the modulus operations, such set membership queries using a Bloom filter constructed with *h* hash functions holding *n* sequences in *m* bits of memory would, for* large m*, have an approximate false positive rate [38] given by

(1) $$\begin{array}{c} f={\left(\;1-e^{-hn/m}\;\right)}^{h}. \end{array}$$

For a fixed target number of sequences in the filter and fixed memory size, the optimal number of hash functions can be calculated as

(2) $$\begin{array}{c} h^{*}=\left\lceil \;\frac{m}{n}\ln2\;\right\rceil . \end{array}$$

Assuming the optimum number of hash functions to be 4, it is feasible to store the human genome in a Bloom filter with a false positive rate of *f* = 6.25% using ca. 2 GB of memory.

Conventional methods (e.g., CityHash from Google, Mountain View, CA) for storing *h* coordinates associated with a given sequence in the memory block use *h* different hash seeds and one hash function. (The term hash seed here refers to an initialization value for the hash function to randomize the distribution of generated hash values and should not to be confused with spaced seeds.) As an alternative, we propose the following.

Let *S*_2*k*_ be a string of length 2*k* over the alphabet Σ = [*A*, *C*, *G*, *T*] represented using a 2-bit encoding with the alphabet mapped to [00,01,10,11], such that complement bases correspond through a bitwise NOT operation. Next, let sequence *S*_2*k*_ (*a* : *b* : *c*) be a substring of *S*_2*k*_ starting from the *a*th letter, ending at the *c*th letter, sampling every *b*th letter, using an index origin of 1. Given a hash function, *H*{·}, we calculate the following four hash values:

(3) xLHS2k1:1:k⊕S2k′1:1:k,xR=HS2kk+1:1:2k⊕S2k′k+1:1:2k,xO=HS2k1:2:2k−1⊕S2k′1:2:2k−1,xE=HS2k2:2:2k⊕S2k′2:2:2k,

where the substring operation is performed prior to reverse complementation denoted by “′” and ⊕ denotes the bitwise XOR operation. The four values calculated in (3) can be interpreted as the left, right, odd, and even hash values for the string *S*_2*k*_. When this string is a concatenation of two *k*-mers, the left and the right hash values will represent the first and the second *k*-mers, respectively, while the odd and even hash values will stretch through the concatenated sequence.

### 3.3. Counting Bloom Filter

In a sequencing experiment, it is often desirable to count the multiplicity of observed sequences. This may be valuable information for removing experimental noise in* de novo* assembly of sequencing data. In RNA-seq experiments, *k-*mer counts may be used to quantitate gene expression levels. Likewise, in metagenome sequencing experiments, they may be used for sequence clustering of similar *k*-mer coverage levels.

However, in almost all applications, the exact count does not necessarily indicate precise abundance of a sequence in the input material, as the shotgun sequencing represents a statistical sampling process. Further, if a short integer counter is used, it may saturate rapidly. On the other hand, if a long integer counter is used, it may inflate memory usage.

Building on the spaced seeds Bloom filter described above, we designed a* staged *Bloom filter with a minifloat counter, as described below.

**Figure 2:**
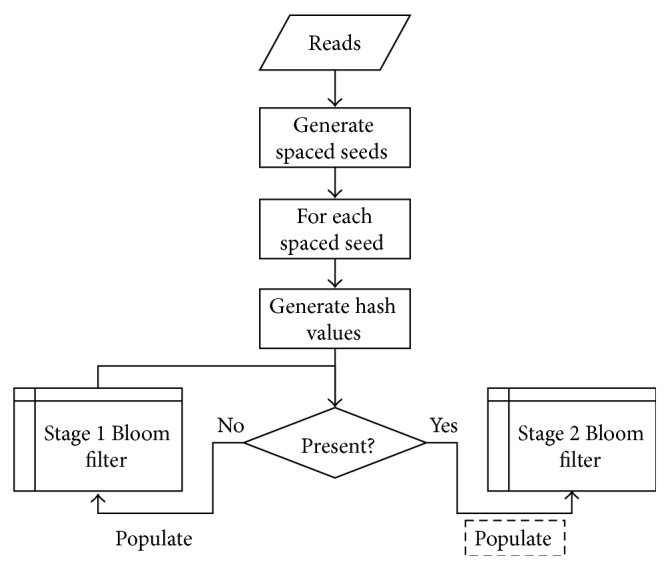
Flowchart of cascading Bloom filters. The process of populating the stage 2 Bloom filter, indicated by the dashed box, is described in Table 1.

When populating the spaced seeds Bloom filter, if the inserted sequence has already been observed (i.e., all the corresponding bits are set in the memory before populating them with the present observation) a pair of hash values are calculated for the concatenated sequence *S*_2*k*_ and its reverse-complement *S*_2*k*_′. These hash values are collapsed by a modulus operation to point to the memory coordinates of a byte array *x* and *x*′, for *S*_2*k*_ and *S*_2*k*_′, respectively. Note that this is a deviation from the regular Bloom filters, which are* bit* arrays, as opposed to* byte* arrays. Also note that *x* and *x*′ are two coordinates on the same byte array. In this construct, the “first-stage” Bloom filter of the spaced seeds records all observed *S*_2*k*_, while the “second-stage” counting Bloom filter is engaged when it is observed at least twice (Figure 2).

At the second stage, the minifloat counter follows an IEEE 754 inspired standard to represent floating point numbers in one byte, the sign standing for the strand information of the recorded sequence. By default, we populate the counter in the direction of the first observed strand. That is, if the positions *x* and *x*′ are both vacant, this would indicate that we have* cascaded* this observed sequence to the second stage for the first time. Assuming that this is not a false positive hit in our spaced seed Bloom filter, the count of observation of this sequence should be two; accordingly the position *x* is populated with a count of 2.

If only one of the positions is nonzero, that position is incremented by one. If it happens to be the coordinate of the reverse-complement sequence, *x*′, then the sign bit is set to indicate that the sequence has been observed on both strands.

If both positions are nonzero, this indicates hash collision. The number negative zero is reserved as a flag for such cases, and both counts are truncated to this special flag. Note that, to avoid secondary hash collisions, that is, hash collisions due to some other pair of sequences, the nonzero condition for this case includes negative zero as well.  Table 1 provides a summary of rules on how the counts are performed.

For the counts, we use a minifloat representation with one sign bit, four exponent bits, and three mantissa bits, with an exponent bias of -2 (or a 1.4.3.-2 minifloat) using the IEEE 754 standard. This gives us exact counts up to 15 and a probabilistic count beyond 16, up to a maximum of 122,880. The precision in the lower end is valuable to control for noise in experimental data. The dynamic range of over five orders of magnitude is conducive to analyzing data from RNA-seq experiments.

**Figure 3:**
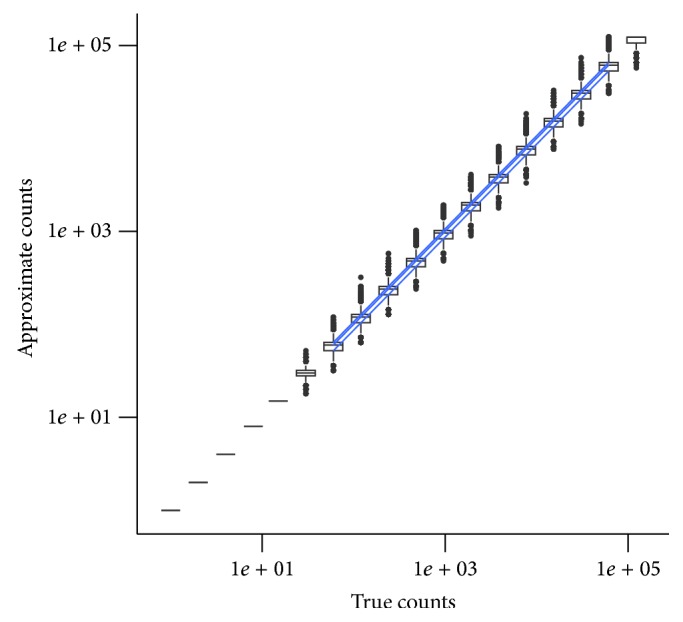
Approximate counts versus true counts in the minifloat data type 1.4.3.-2. The box-whisker plots indicate the interquartile range and the variability of the counts outside the first and the third quartiles. The distributions represent a repetition of 10,000 counts in each logarithmic bin.

Table 2 illustrates some counts and their minifloat representations.  Figure 3 shows the tight concordance between the approximate counts from probabilistic minifloat values and the true counts.

## 4. Application Areas

### 4.1. Error Correction

Above, we noted that read errors within the space between seeds would not affect the sequence *S*_2*k*_. Further, the Bloom filter with the four hash values *x*_*L*_, *x*_*R*_, *x*_*O*_, and *x*_*E*_ would have certain characteristics when the sequencing data has read errors.

For example, when an interrogated sequence *S*_2*k*_ has a single base error, then the error should be confined to the left or the right half of the sequence. Likewise, it should be confined to the odd or even bases. Therefore, if an error-free version of the sequence is already recorded in the Bloom filter, two out of four hash values should register hits with a particular pattern, such as the left and the odd hash values. If the other two subsequences can be modified by the same one-base change, such that they also register hits, it would be a strong indication that the correct sequence should have been the sequence with this change. This can further be supported through correlating* corrected* bases to their estimated sequencing qualities, such as the *q*-scores generated by the data acquisition software of the sequencing instruments.

Optionally, we can use both the spaced seeds and their counts to guide error correction.  Table 3 summarizes how the error correction may be performed.

### 4.2. Sequence Assembly

The extension of the DBG assembly algorithm using spaced seeds hash table involves minor modifications to the ABySS algorithm [24].

When the assembly of a contig is initiated, it would go through a transient phase with two “wave fronts” corresponding to extensions of the first and the second *k*-mer. After the seed gap is traversed, while the leading edge probes for possible extensions, the lagging edge would eliminate false branches by asserting that the extension on the lagging edge agrees with the assembled sequence between the seeds. Such assertions would realize the benefits of the improved sequence uniqueness of spaced seeds demonstrated in Figure 1.

It is also possible to use the spaced seeds Bloom filter design for sequence assembly, when the data type is supplemented with auxiliary information about the sequences of certain *k*-mers. While building the spaced seeds data type, one can query it at the same time for the presence or absence of 1-base extensions of the populated spaced seeds, as well as their uniqueness. These will indicate whether the *k*-mers under consideration are blunt ends (with no neighbors in one or both directions) or at some branching points in the corresponding DBG. If so, their sequences can be saved as auxiliary data along with their types (blunt or branch). Such sequences can be used to initiate the assembly.

During the construction of the spaced seeds Bloom filter, the list of blunt edges will go through a transitory phase, where initially there will be two blunt edges for each read. As the set of sequences converges to the target assembly size, blunt edges will be reported only at places of low or missing sequence coverage and at the edges of target sequences (as in the chromosome ends for genomes or transcript ends for transcriptomes). Depending on the size of the problem, a periodic “garbage collection” might be necessary, where the list of blunt edges is interrogated again to see if any members in the list have extensions in the populated filter and they are removed from the list if so.

However, branch sequences would not have similar issues. At branching points, it is enough to capture branching using any representative of the branching sequences. While populating the filter, the first sequence to cross a branching point would not be labeled as such, but the second sequence to do so would necessarily be caught and labeled accordingly.

DBG construction using spaced seed sequences is expected to reconstruct all the edges and vertices but would also have extra edges and vertices stemming from false positive hits in the Bloom filter. However, the pattern of false positive branches on the graph would be easily distinguishable using their lengths.

If the probability of a false positive hit in our Bloom filter is *f*, then the probability of this branch to get extended to another false node will be *f* ^2^, and this probability will continue to drop geometrically as the length of the false branch gets longer. This represents a massive multiple hypothesis testing problem, even at the scale of the human genome where the probability of a false branch of length 10 will be very small compared to the number of hypotheses tested.

In our example of *f* = 6.25%, the probability of a false branch of length 10, after a naïve Bonferroni correction with a factor of 3 × 10^9^, is still less than 0.3%. Keeping in mind that the ABySS algorithm already implements a default branch trimming for branches shorter than 2*k* − 1 bases long, branch removal due to false Bloom filter hits will be benign, in comparison.

By design, the spaced seeds Bloom filter harbors information at two length scales: *k* and 2*k* + Δ. As such, the assembly process can potentially switch between these two scales. A smaller length scale is valuable when the local sequence coverage is low, and a large length scale is valuable when the local sequence complexity is low. Being able to dynamically switch between these two length scales would potentially allow the assembly algorithm to navigate its way out of these challenging situations.

Using the coverage information captured in the counting Bloom filter data structure may further strengthen the assembly process. For example, in transcriptome assembly, the abundance of sequences in the experimental data would be indicative of the expression levels of the corresponding transcripts. Using the counting Bloom filter, one can partition the assembly problem into strata of expression levels, constructing sequences across spaced seeds with similar counts. However, the presence of alternatively spliced transcripts would be a challenge that would need to be mitigated. Allowing temporary “excursions” into other coverage strata may help resolve this issue.

Similarly, for metagenome assembly, partitioning the assembly problem into strata of counts would also be a viable approach, albeit with similar caveats. Identical or nearly identical sequences from less abundant species would yield gaps in their assembled genomes, when the higher coverage branches are used exclusively to assemble the genomes of highly abundant species. As is the case for transcriptome assemblies, count stratification can be performed taking the graph topology into account and holds the potential to disambiguate and catalog sequences with uneven representation.

## 5. Conclusions

The de Bruijn graph has reigned for over a decade as the data type of choice for short read assembly algorithms but could be supplanted by other paradigms as its advantages are becoming less apparent with increased read lengths. Here, we present novel data types that will help de Bruijn graphs maintain their competitive edge over alternatives. Specifically, we generalize the definition of a *k*-mer, the traditional workhorse of de Bruijn graphs, to a “spaced seed”: a *k*-mer pair, consisting of two shorter sequences separated by a fixed distance, Δ.

The concept can be generalized for any topology of the spaced seed templates, such as those used for sequence alignments [37], but the special case we use is more amenable to de Bruijn graph applications. When a spaced seed is extended within a de Bruijn graph, 1-base extensions at all the match/do not care transitions and the last matched base (if the latter corresponds to the template edge) need to be tracked. In our design, that is two bases per spaced seeds. Further, the design has the potential to be extended to represent paired end reads, when Δ is variable.

We introduce three data types to represent the spaced seeds. The first one is an extension of the *k-*mer hash table in the ABySS algorithm. The second one uses a specialized Bloom filter data structure to store them implicitly. Lastly, we describe the design of a counting Bloom filter in association with a minifloat counter to represent the abundance of *k*-mers deterministically up to 15-fold and store higher abundances in a probabilistic fashion.

Although the advantages of a Bloom filter as a low memory footprint alternative to a hash table have been reported before [32], extending the concept to include spaced seeds leverages their efficiency to report additional true positive sequences across longer distances, potentially offering longer range contiguity for* de novo* sequence assemblies. Also, the designed spaced seed data types that use Bloom filter data structures support sequence contig construction at two length scales (*k* and 2*k* + Δ), a feature expected to handle low coverage and sequence complexity issues. Further, we note a major advantage of the designed data types in their potential utility for sequence error correction.

Another potential use case for spaced seeds is in the problem of assembly scaffolding. We have implemented an algorithm, LINKS [39], which takes long reads, such as those generated by the Oxford Nanopore MinION sequencer to scaffold or rescaffold draft assemblies. It uses a hash table of *k*-mer pairs to register linkage information between assembled contigs or scaffolds. Weighing the evidence of *k-*mer positions on linked contigs or scaffolds and frequencies of linkage observation, it constructs paths through the linked sequences to build scaffolds.

For algorithmic complexities of various use cases, although we expect the major impact of the spaced seeds to be on reduced memory footprints, we note that reduced space complexity should result in reduced run times. That is, even if the time complexity of the algorithms as a function of the input data would not change, their scaling constants would. This behavior would be mostly due to the memory management and cache performance of the affected algorithms.

The spaced seed data type concepts proposed herein will help us retain the succinct representation advantages of de Bruijn graphs for sequence assembly of data from high throughput sequencing technologies and carry the paradigm forward to the era of long reads, when it arrives.

## Figures and Tables

**Table 1:** Update rules for the counting Bloom filter.

Value at bit location		Update action		
*x*	x′	At location	Set sign	Set count
0	0 or −0	x	0	2
Nonzero	0 or −0	x	No change	Increment
0 or −0	Nonzero	x′	1	Increment
Nonzero	Nonzero	x and x′	1	0
−0	0	x′	1	2

**Table 2:** Minifloat counts and their representations.

Count (*c*)	Mantissa^a^ (*t*)	Exponent^a^ (*e*)
Zeros		
0 and −0^b^	000	0000

Subnormal numbers^c^ (*c* = *t*)		
1	001	0000
2	010	0000
⋮	⋮	⋮
7	111	0000

Normalized numbers^c^ (*c* = 1 · *t* × 2^*e*+2^)		
8	000	0001
9	001	0001
⋮	⋮	⋮
15	111	0001
16	000	0010
18	001	0010
⋮	⋮	⋮
122,880^d^	111	1110

^a^Most significant digits on the left.

^b^Distinguished by the sign bit.

^c^Shown for a sign bit of 0.

^d^Maximum possible 1.3.4.-2 minifloat number.

**Table 3:** Error correction rules.

Value at bit location				Interpretation	Action
*x*_*L*_	*x*_*R*_	*x*_*O*_	*x*_*E*_		
**1**	**1**	**1**	**1**	Present in the set	Update count

0	0	0	0	Not present in the set	Insert in the filter
**1**	**1**	**1**	0		
**1**	**1**	0	**1**		
**1**	0	**1**	**1**		
0	**1**	**1**	**1**		

**1**	0	**1**	0	There may be a single base correction that would make the pattern (1111)	If so, and if the corrected sequence has a nonzero count, correct the read. If not, insert in the filter.
**1**	0	0	**1**		
0	**1**	**1**	0		
0	**1**	0	**1**		
				
**1**	0	0	0	There may be two base corrections that would make the pattern (1111)	
0	**1**	0	0		
0	0	**1**	0		
0	0	0	**1**		
